# Book Reviews

**Published:** 1912-12

**Authors:** 


### BOOK REVIEWS

AN INTRODUCTION TO THE STUDY OF INFECTION AND IMMUNITY. INCLUDING SERUM THERAPY, VACCINE THERAPY, CHEMOTHERAPY AND SERUM DIAGNOSIS. By Charles E Simon, M. D., Professor of Clinical Pathology and Experimental Medicine, College of Physicians and Surgeons, Baltimore. Octavo, 301 pages; illustrated. Cloth $3.25 net. Lea & Febiger, Publishers, Philadelphia and New York, 1912.

This work is an exposition of one of the most recent, most important, and most promising developments which have thus far occurred in medical science, namely, the laboratory revelation of the processes by which the human organism protects itself from disease, or if the defence is overcome, by which the infections establish themselves. The results of the study of this subject are so far reaching and of such extreme practical value that every practitioner should familiarize himself not only with the essential basis upon which the new science of immunology has been established, but also with its application to diagnosis and treatment.

---

A TREATISE ON PELLAGRA. By Edward Jenner Wood, S. B., M. D., Chairman of the Pellagra Commission, North Carolina Board of Health; Member of the American Society of Tropical Medicine; Fellow of the London Society of Tropical Medicine and Hygiene; Formerly President of the Medical Society of North Carolina, etc. D. Appleton & Co., New York and London.

In this work the student of pellagra will find a most valuable abstract of the foreign and American literature. The chapter on History and Georgraphical Distribution is as complete as it can well be. Theories of Etiology are presented in an unbiased manner, both sides of the question are freely discussed by Dr. Wood. The chapters on Symptoms and Diagnosis will be valuable to the practitioner. The work is well illustrated. Careful study of these chapters will be a great aid to those who have not become familiar with the symptom complex. It is worthy of note that American writers almost as a whole are taking a much more hopeful attitude toward the prognosis and treatment of the disease. The present work though not so optomistic as some, is somewhat encouraging. Dr. Wood's method of treatment will prove a help to a great many, though it is to be hoped that his conclusions in regard to two of the most distressing symptoms, viz. the diarrhoea and stomatitis, will not deter others in the use of remedies which may have found to be effectual.

---

A MANUAL OF AUSCULTATION AND PERCUSSION, EMBRACING THE PHYSICAL DIAGNOSIS OF DISEASES OF THE LUNGS AND HEART, AND OF THORACIC ANEURYSM, AND OF OTHER PARTS. By Austin Flint, M. D., LL. D., Late Professor of Medicine and Clinical Medicine in the Bellevue Hospital Medical Colleg, etc., New York. Revised by Haven Emerson, A. M., M. D., Associate in Physiology and in Medicine, College of Physicians and Surgeons, Columbia University, New York. 12mo, 361 pages, illustrated. Cloth, $2.00 net. Lea & Febiger, Philadelphia and New York, 1912.

The revision and republication of this classic should be a source of gratification to all American physicians, for since its original appearance it has been recognized as a masterpiece of clarity and precision by one of the greatest of American teachers. The importance of variations in pitch, which was Flint's own discovery, was explained by him in the simplest manner. By a strict adherence to facts, he gave to his writings an enduring worth, so that it has been possible, with but few changes, to bring his *Ausculation and Percussion* up to the present state of science. In this new edition the scope of the book has been widened by the inclusion of chapters on the examination of the abdominal viscera and of the nervous system.

---

A TEXT-BOOK UPON THE PATHOGENIC BACTERIA AND PROTOZOA. For Students of Medicine and Physicians. By Joseph McFarland, M.D., Professor of Pathology and Bacteriology in the Medico-Chirurgical College, Philadelphia. Seventh edition, thoroughly revised. Octavo of 878 pages, 293 illustrations, a number of them in colors. Philadelphia and London: W. B. Saunders Co., 1912. Cloth, $3.50.

This book covers well the subject of Bacteria and Protozoa. not being a mere handbook, but covering the subject to the present time fully. It does not stop with the organisms, themselves, but takes up their toxins, their characters and individuality. The section devoted to immunity is very plain indeed. The reader is not lost as it were in theories, but as he passes from pages to page the clearness of this book and importance of this branch of medicine impresses him.

---

DISEASES OF THE GENITO-URINARY ORGANS AND THE KIDNEY. By Robert H Greene, M. D., Professor of Genito-Urinary Surgery at the Fordham University, New York; and Harlow Brooks, M. D., Assistant Professor of Clinical Medicine, University and Bellevue Medical College. Third Revised Edition. Octavo of 639 pages, 339 illustrations. Philadelphia and London, W. B. Saunders Company, 1912. Cloth, $5.00 net; Half Morocco, $6.50 net.

This well known book has been revised to date and affords a valuable guide in the surgical proceedings recommended. Cystospy and modern tests for the renal function are given ample space. These authors have given much study to the subject of prostatic enlargement of elderly men. The reviewer would like for them to study the secretion from mild chronic prostatitis with the dark field illumination and dove-tail their findings with the facts they have already recorded as to the pathology. The myriads of organisms they will often find, (with practically no pus) and the toxins produced by them, will afford a connecting link which is lacking in their theory.

---

A TEXT-BOOK ON THE PRACTICE OF GYNECOLOGY. For Practitioners and Students. By W. Easterly Ashton. M. D., LL. D., Professor of Gynecology in the Medico-Chirurgical College of Philadelphia. Fifth Edition, Thoroughly Revised. Octavo of 1100 pages, with 1050 original line drawings. Philadelphia and London: W. B. Saunders Company, 1912. Cloth, $6.50 net; Half Morocco, $8.00 net.

The first edition of this splendid work was published in 1805, and since then five editions and five reprints have been issued. This fact shows the demand for a book on gynecology written with the idea of taking nothing for granted in describing diseases and their treatment. The revision has been thorough and brings the work fully up to date in every respect. Many additions have been made to the chapter on X-ray. Additions have been made to palliative treatment of cancer of the uterus and vagina, and the use of actetone and sulphate of quinine advised. The recent advances in the diagnosis and treatment of syphilis have been carefully considered and special reference made to the excision of the primary lesion and the use of salvarsan.

---

A MANUAL OF AUSCULTATION AND PERCUSSION. By Austin Flint, M. D., LL. D., Late Professor of Medicine and of Clinical Medicine in the Bellevue Hospital Medical College, etc., New York. Revised by Haven Emerson, A. M., M. D., of the College of Physicians and Surgeons, Columbia University, New York, New (6th) edition revised and enlarged. 12mo. 354 pages; illustrated. Cloth $2 net.

This little book affords an epitome of the subject which greatly simplifies it for students and practitioners of medicine.

A TEXT-BOOK OF OBSTETRICS. By Barton Cooke Hoist, M. D., Prof. of Obstetrics in the University of Pennsylvania. Seventh Edition revised and enlarged with 895 illustrations—53 in colors. Pp. 1013. W. B. Saunders Co., Phladelphia.

This classical work has been thoroughly revised and contains the best there is in medical literature on obstetrics. The profuse illustrations teach quickly and well many facts that would require hours of reading and study to secure. The wide experience of the author has ably fitted him for the task of writing such a book, which reflects his paintaking care and ability throughout.

---

FRACURES AND DISLOCATIONS. — A PRACTICAL TREATISE ON FRACTURES AND DISLOCATIONS. By Lewis A. Stimson, B. A., M. D., Professor of Surgery in Cornell University Medical College, New York. 7th edition. Octavo 930 pages, with 459 engravings and 39 plates. Cloth, $5.00 net. Lea & Febiger, Philadelphia.

The demand for seven editions of this book attests its value. Dr. Stimson has long been recognized as the greatest living authority on fractures and dislocations and his book is acknowledged to be one of the most complete and satisfactory in the English language. The principal additions to this edition have been made in connection with the subject of treatment, especially in that of old dislocations and in respect to the operative treatment of recent fractures. More than 100 new illustrations have been added.

---

A CYCLOPEDIA OF AMERICAN MEDICAL BIOGRAPHY Comprising the Lives of Eminent Deceased Physicians and Surgeons from 1610 to 1910. By Howard A. Kelly, M. D. Illustrated with Portraits. Volume II, Pp. 545. W. B. Saunders Co., Philadelphia.

Kelly presents much interesting and inspiring information concerning the pioneers and prominent men in medicine and surgery.

A TREATISE ON DISEASES OF THE HAIR, by Geo. T. Jackson, M. D., Prof. of Dermatology in the College of Physicians and Surgeons, New York, and Chas. W. McMurtry, M. D., Instructor in Dermatology at the same college. Illustrated with 109 engravings and 10 colored plates. Lea & Febiger, Philadelphia.

The helplessness of the average physician who is confronted with the treatment of a disease of the scalp is too well known to us all. Jackson and McMurtry have brought together in this comprehensive work all that is known about such diseases. Not only will such a work remove many erroneous ideas now prevalent, but it will afford a valuable help in the diagnosis and treatment of diseases of the scalp. This book contains the most complete presentation of ring worm of the scalp and forms yet presented, except in the works of Sabourand. The illustrations are excellent.

---

ELEMENTARY BACTERIOLOGY AND PROTOZOOLOGY. The microbiological causes of infectious diseases, by Herbert Fox, M. D. Director in the William Pepper Laboratory of Chemical Medicine in the University of Pennsylvania. Illustrated with 67 engravings and 5 colored plates. Lea and Febiger, Philadelphia.

Much technical material has been omitted from this work, especially in the subject of biological differentiation. Emphasis has been laid upon how bacteria pass from individual to individual, how they enter the body and act when once within, and their manner of exit. It is a splendid little book.

---

THE SURGICAL CLINICS OF JOHN B. MURPHY, at the Mercy Hospital, Chicago, Ill. June 1912. Published monthly by W. B. Saunders Co., Philadelphia. Price per year $8.00.

Mention has been made previously to these condensed medical works. A careful study of the mshould broaden a surgeon's view point and at the same time improve his surgical judgment and technic.

THE SURGICAL CLINICS OF JOHN B. MURPHY, M. D.. at Mercy Hospital, Chicago. Volume I. Number IV., (August). Octavo of 154 pages, illustrated. Philadelphia and London: W. B. Sanders Company, 1912. Published Bi-Monthly. Price per year: Paper, $8.00. Cloth $12.00.

Among the interesting subjects in this number are acute appendicitis and pneumonia; chronic appendicitis ankylosis of the knee; angiophlebitis; hypertrophy of the prostate; nephropyleplasty; traumatic epilepsy; transplantation of bone; and carcinoma of the splnic --exure of the colon.

---

COLLECTED PAPERS BY THE STAFF OF ST. MARY'S HOSPITAL (MAYO CLINIC FOR 1911. Octavo of 603 pages, illustrated. Philadelphia and London: W. B. Saunders Company, 1912. Cloth, $5.50 net.

We have previously revised in the Journal-Record other volumes of collected papers from the Mayo-Clinic. Take pleasure in looking over the excellent one embodied in this book. They cover a wide range of important subjects.

---

PROGRESSIVE MEDICINE—A QUARTERLY DIGEST OF ADVANCES, DISCOVERIES AND IMPROVEMENTS IN THE MEDICAL AND SURGICAL SCIENCES, COVERING THE ENTIRE DOMAIN OF PRACTICAL MEDICINE. Edited by Hobart Amory Hare, M. D., Professor of Therapeutics, Jefferson Medical College, Philadelphia. Sept. 1912. Price, bound in heavy paper, $6.00 per annum; bound in cloth, $9.00 per annum, postpaid. Lea & Fiebiger, Philadelphia.

The September number of this valuable digest of current medical literature is well up to its usual standard of excellence.

---

A COLLECTION OF PAPERS, Published to 1908, by W. J. Mayo, M. D., and C. H. Mayo, M. D., Rochester, Minn. Volume II, Pp. 591. Published by W. B. Saunders Co., Philadelphia.

The first volume of this work was revised sometime ago and affords an easy form in which to preserve the writings of these phenomenal men. The wide variety of subjects discussed and the authoritative manner in which the surgical procedures are described.

